# Engineering marine fungi for conversion of d-galacturonic acid to mucic acid

**DOI:** 10.1186/s12934-020-01411-3

**Published:** 2020-07-31

**Authors:** Virve Vidgren, Satu Halinen, Anu Tamminen, Susanna Olenius, Marilyn G. Wiebe

**Affiliations:** VTT Technical Research Centre of Finland Ltd, Tietotie 2, P.O. Box 1000, 02044 Espoo, Finland

**Keywords:** d-galacturonic acid, Galactaric acid, Mucic acid, Marine fungi, CRISPR, Synthetic expression system

## Abstract

**Background:**

Two marine fungi, a *Trichoderma* sp. and a *Coniochaeta* sp., which can grow on d-galacturonic acid and pectin, were selected as hosts to engineer for mucic acid production, assessing the suitability of marine fungi for production of platform chemicals. The pathway for biotechnologcial production of mucic (galactaric) acid from d-galacturonic acid is simple and requires minimal modification of the genome, optimally one deletion and one insertion. d-Galacturonic acid, the main component of pectin, is a potential substrate for bioconversion, since pectin-rich waste is abundant.

**Results:**

*Trichoderma* sp. LF328 and *Coniochaeta sp*. MF729 were engineered using CRISPR-Cas9 to oxidize d-galacturonic acid to mucic acid, disrupting the endogenous pathway for d-galacturonic acid catabolism when inserting a gene encoding bacterial uronate dehydrogenase. The uronate dehydrogenase was expressed under control of a synthetic expression system, which fucntioned in both marine strains. The marine *Trichoderma* transformants produced 25 g L^−1^ mucic acid from d-galacturonic acid in equimolar amounts: the yield was 1.0 to 1.1 g mucic acid [g d-galacturonic acid utilized]^−1^. d-Xylose and lactose were the preferred co-substrates. The engineered marine *Trichoderma* sp. was more productive than the best *Trichoderma reesei* strain (D-161646) described in the literature to date, that had been engineered to produce mucic acid. With marine *Coniochaeta* transformants, d-glucose was the preferred co-substrate, but the highest yield was 0.82 g g^−1^: a portion of d-galacturonic acid was still metabolized. *Coniochaeta* sp. transformants produced adequate pectinases to produce mucic acid from pectin, but *Trichoderma* sp. transformants did not.

**Conclusions:**

Both marine species were successfully engineered using CRISPR-Cas9 and the synthetic expression system was functional in both species. Although *Coniochaeta* sp. transformants produced mucic acid directly from pectin, the metabolism of d-galacturonic acid was not completely disrupted and mucic acid amounts were low. The d-galacturonic pathway was completely disrupted in the transformants of the marine *Trichoderma* sp., which produced more mucic acid than a previously constructed *T. reesei* mucic acid producing strain, when grown under similar conditions. This demonstrated that marine fungi may be useful as production organisms, not only for native enzymes or bioactive compounds, but also for other compounds.

## Background

Marine-derived fungi are considered an under-utilized resource, with particularly high potential for supply of bioactive compounds [1–3], but also supplying novel enzymes [4] or functioning as whole biocatalysts [5]. Their ability to tolerate high salt concentrations may also be beneficial in production of other metabolites, such as platform chemicals which are needed in high concentrations, although genetic modification of marine fungi has focused on enhancing secondary metabolite production [6]. Having observed in a recent student study of carbohydrate utilization by diverse fungi isolated from marine environments [7] that many of these grew on both d-galacturonic acid and pectin, we wanted to assess whether some of these fungi could be genetically engineered and could then produce the platform chemical mucic (galactaric) acid.

Mucic acid was selected as the chemical to be produced in this study because the pathway for its synthesis generally requires minimal engineering in filamentous fungi [8, 9], which would be beneficial when working with fungi for which no genome data was available. As a chemical, mucic acid has current applications in some skin care products, e.g. by Yves Rocher [10], Melvita [11], Novexpert [12], and Annemarie Börlind [13], as a chelator, but it also has potential use as a precursor for polymer synthesis. Mucic acid can be reduced to adipic acid [14], a precursor for nylon, and can be converted to furan-2,5-dicarboxylic acid (FDCA). FDCA is of interest since it can substitute for fossil-based terephthalic acid (PTA) in the production of polyesters and other polymers containing an aromatic moiety [15].

Biotechnologically, mucic acid is produced from d-galacturonic acid, which is derived from pectin. Citrus pulp, from orange juice production, and sugar beet pulp, remaining after sugar extraction, are both pectin-rich (containing 20–30% pectin, of which ~ 70% is d-galacturonic acid, [16, 17] wastes that are widely available worldwide. These wastes are potential raw material sources for the production of mucic acid. Conversion of d-galacturonic acid to mucic acid utilizes the activity of uronate dehydrogenase (udh, see Additional file 1: Fig. S1), an enzyme present in some bacteria. Different microbes, such as the yeast *Saccharomyces cerevisiae* [18], the bacterium *Escherichia coli* [4, 19] and two filamentous fungi *Aspergillus niger* [9] and *Trichoderma reesei* [8, 20] have been engineered for the conversion of d-galacturonic acid to mucic acid by introducing the bacterial *udh* gene to these organisms. Udh requires NAD^+^ as co-factor [21]. Provision of a co-substrate such as d-glucose or lactose has been shown to improve mucic acid production, providing carbon for biomass, energy and options for redox balancing [18, 19, 22].

In addition to introducing the *udh* gene to an organism lacking it, the endogenous pathway for d-galacturonic acid metabolism needs to be inactivated. In the fungi which have been studied, there is a three enzyme pathway for d-galacturonic acid utilization (see Additional file 1: Fig. S1) [23–25]. In this reductive pathway, d-galacturonate is reduced to l-galactonate by a d-galacturonate reductase [23]. l-Galactonate dehydratase and 2-keto-3-deoxy-l-galactonate aldolase then convert the l-galactonate to pyruvate and glycerol [23]. It has been shown that deletion of the first enzyme of the pathway, d-galacturonate reductase, is sufficient to shut down the whole pathway [8]. However, two different d-galacturonate reductase encoding genes, *gar1* and *gar2* (or *gaaA*), have been identified among filamentous fungi, and either or both may be responsible for d-galacturonate reductase activity [24–26].

Since no information was available about gene expression and regulation in the selected marine fungi, a recently developed synthetic expression system (SES) that was shown to function in a wide spectrum of fungal species [27] was used to express the *udh*. The system is composed of two expression cassettes. The first cassette expresses the synthetic transcription factor (sTF) at a constitutive low level, using a yeast-derived core promoter that has been validated for a variety of organisms [27]. The second cassette expresses the target gene under an sTF-controlled synthetic promoter [27]. Binding of the synthetic transcription factor to the synthetic promoter causes strong and constitutive expression of the target gene without need for an endogenous promoter, transcription regulators or activators.

Initially six marine fungi that were able to grow on d-galacturonic acid and pectin were selected for the study. The ability to grow on pectin as well as d-galacturonic acid suggested that these strains secreted pectinases, as well as having good uptake of d-galacturonic acid. Production of pectinases and other hydrolytic enzymes could be useful in developing a consolidated bioprocess, i.e. hydrolysis and conversion of pectin rich plant biomass to mucic acid by the producing organism. *Trichoderma* sp. LF328 and *Coniochaeta* sp. MF729 were the only fungi out of the six, from which the d-galacturonic acid reductase gene could be identified. These marine fungi grew well at low pH, which has been shown to be good for mucic acid production with *T. reesei* [22] and *S. cerevisiae* [18].

The aim of the present study was to construct marine *Trichoderma* and *Coniochaeta* strains that produce mucic acid. An expression cassette containing a *udh* gene under the control of SES was integrated in the genomes of marine *Trichoderma* sp. LF328 and *Coniochaeta* sp. MF729. This was achieved with CRISPR/Cas9 in such a way that the endogenous pathway for d-galacturonic acid catabolism was disrupted at the same time that the expression cassette was integrated to the locus. The applicability of the SES in marine fungi was assessed for the first time and was shown to work efficiently. Transformants were obtained from both strains and mucic acid was produced from d-galacturonic acid, poly-d-galacturonic acid or pectin in small-scale (24-well plates) cultivations. Mucic acid was also produced in 1.5 L bioreactors, to further assess the production ability of the best strain with three potential co-substrates and controlled pH, and to determine whether a constant pH environment would improve the ability of the best *Coniochaeta* transformant to convert pectin to mucic acid.

## Results

### *gar1* and *gar2* amplification with degenerative primers

Marine *Trichoderma* sp. LF328 and *Coniochaeta* sp. MF729 were able to metabolize d-galacturonic acid and thus should possess *gar1* or *gar2*, or both. There was no sequence data available in gene banks for either marine *Trichoderma* or *Coniochaeta* species to verify the presence of these genes. Thus sequence data from other relevant species, e.g. filamentous fungi and yeast, were used to design degenerative primers for *gar1* and *gar2* amplification from LF328 and MF729.

With *gar*1 degenerative primers a fragment was amplified with the control *T. reesei* QM6a as a template, which was verified to be *gar1* by sequencing. However, with LF328 or MF729 as templates no fragment was amplified. With *gar2* degenerative primers, a putative d-galacturonic acid reductase was amplified with all three templates, LF328, MF729 and *T. reesei* QM6a. These fragments were sequenced and produced readable sequence of 416 nt for MF729, 498 nt for LF328 and 539 nt for *T. reesei*. As expected, the fragment that was amplified using control *T. reesei* QM6a as a template was 100% identical to the gene bank *T. reesei gar2* sequence. The MF729 and LF328 fragments had 83% and 82% identity to the *T. reesei gar2*, respectively. In comparison, the MF729 and LF328 fragments had only 52% and 53% sequence identity, respectively, to the *T. reesei gar1*. Based on these results *gar2* was the only gene to be deleted in order to destroy the endogenous d-galacturonic acid pathway in these two marine strains. The *gar2* sequences obtained for LF328 and MF729 (see Additional file 1: Fig. S2) were used to design the protospacers for CRISPR-Cas9 used in genetic modification of the strains. The 3′ and 5′ flanks for cassette integration to the genome were also planned based on the *gar2* sequences. Short flanking sequences were used for integration, from 0.13 to 0.26 kb. Two transformants, both with correct integration, were obtained from *Trichoderma* sp. LF328 and 34 transformants from *Coniochaeta* sp. MF729. Not all *Coniochaeta* transformants were screened for correct integration: but two out of three that were screened showed correct integration.

### Production of mucic acid from d-galacturonic acid in 24-well plates

**Fig. 1:**
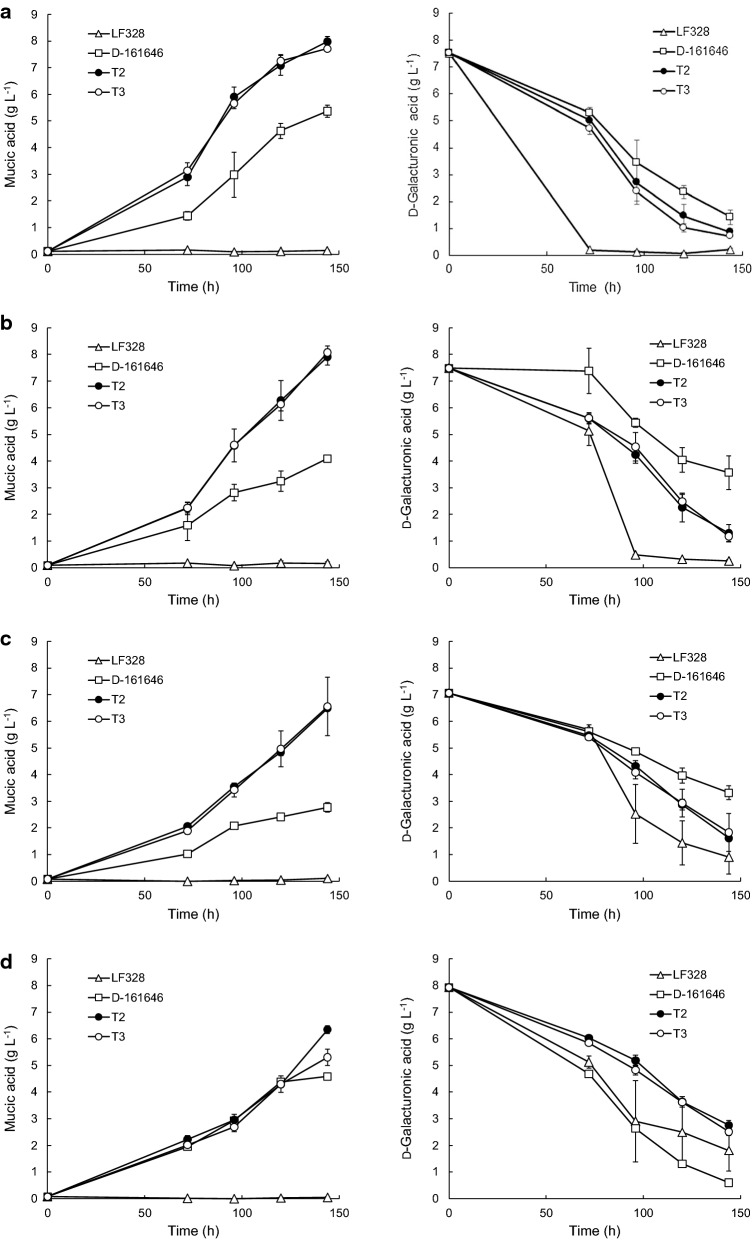
Mucic and residual d-galacturonic acid concentrations in 24-well plate cultures of *Trichoderma* sp. transformants. *Trichoderma* sp. LF328 and its transformants T2 and T3 were grown in 4 ml medium containing pure d-galacturonate and lactose (**a**), d-xylose (**b**), d-glucose (**c**) and glycerol (**d**) as co-substrate. *T. reesei Δgar1 udh* strain D-161646 was included as a positive control. Error bars represent ± standard error of the mean for n = 3 biological replicates

**Fig. 2:**
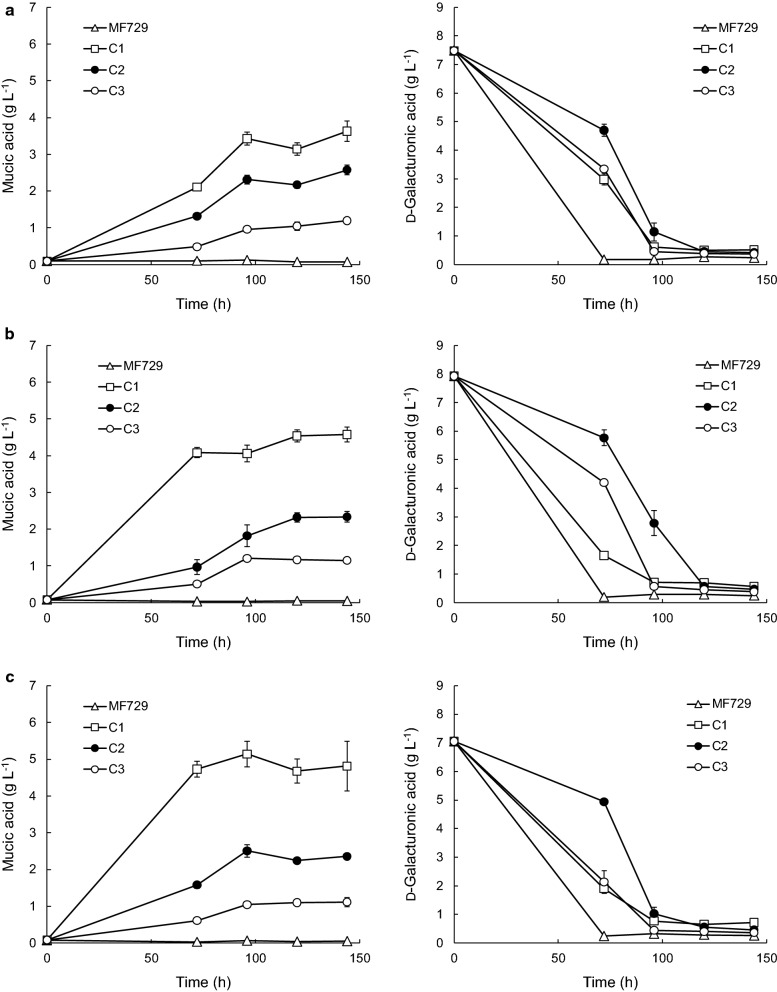
Mucic and residual d-galacturonic acid concentrations in 24-well plate cultures of *Coniochaeta* sp. transformants. *Coniochaeta* sp. MF729, its transformants C1, C2 and C3 were grown in 4 ml medium containing pure d-galacturonate and d-xylose (**a**), d-glucose (**b**) and glycerol (**c**) as co-substrate. Error bars represent ± standard error of the mean for n = 3 biological replicates

Two marine *Trichoderma* transformants, T2 and T3, and three *Coniochaeta* transformants, C1, C2 and C3, in which the *udh* gene was expressed under the control of SES-B and the endogenous d-galacturonate pathway had been disrupted, were cultivated in 24-well plates, with *T. reesei* D-161646, the earlier constructed strain which has produced up to 21 g L^−1^ mucic acid in fed-batch culture, as a positive control [8, 20, 22]. All five transformants produced mucic acid from d-galacturonic acid, regardless of the co-substrate, lactose (*Trichoderma* strains only), d-glucose, d-xylose or glycerol (Figs. 1 and 2; Table 1) [28]. *Coniochaeta* and its transformants did not grow on lactose. Neither MF729 nor LF328 produced mucic acid (Figs. 1 and 2), although both strains consumed d-galacturonic acid in all media.

**Table 1:** Mucic acid production characteristics of marine fungi in 24-well plates

Strain	T2	T3	D-161646	C1	C2	C3
Lactose						
Mucic acid (g L^−1^)	8.0 ± 0.2 ^aA^	7.7 ± 0.0 ^aB^	5.4 ± 0.2 ^bA^			
Yield mucic acid/d-galacturonic acid (g g^−1^)	1.20 ± 0.05 ^a^	1.13 ± 0.00 ^a^	0.88 ± 0.05^b^			
d-Xylose						
Mucic acid (g L^−1^)	7.9 ± 0.3^aA^	7.8 ± 0.2^aB^	4.1 ± 0.1^bB^	3.4 ± 0.2^aA^	2.3 ± 0.1^bA^	1.0 ± 0.0^cA^
Yield mucic acid/d-galacturonic acid (g g^−1^)	1.28 ± 0.05^a^	1.29 ± 0.08^a^	1.11 ± 0.18^a^	0.50 ± 0.03^a^	0.37 ± 0.01^b^	0.14 ± 0.01^c^
Glycerol						
Mucic acid (g L^−1^)	6.4 ± 0.2^aB^	5.3 ± 0.3^bA^	4.6 ± 0.1^cB^	4.5 ± 0.2^aB^	2.3 ± 0.1^bA^	1.2 ± 0.6^cA^
Yield mucic acid/d-galacturonic acid (g g^−1^)	1.23 ± 0.02^a^	0.98 ± 0.04^b^	0.63 ± 0.02^c^	0.57 ± 0.02^a^	0.29 ± 0.02^b^	0.16 ± 0.01^c^
d-Glucose						
Mucic acid (g L^−1^)	6.5 ± 0.1^aB^	6.6 ± 1.1^aAB^	2.8 ± 0.2^bC^	5.1 ± 0.4^aB^	2.5 ± 0.2^bA^	1.1 ± 0.1^cA^
Yield mucic acid/d-galacturonic acid (g g^−1^)	1.19 ± 0.01^a^	1.24 ± 0.05^a^	0.75 ± 0.09^b^	0.82 ± 0.05^a^	0.42 ± 0.02^b^	0.17 ± 0.01^c^

Marine *Trichoderma* sp. LF328 transformants (T2 and T3) and *Coniochaeta* sp. MF729 transformants (C1, C2 and C3) were grown in 24-well plates with d-galacturonic acid as substrate and lactose, d-xylose, glycerol or d-glucose as co-substrate. *T. reesei Δgar1 udh* strain D-161646 was used as a positive control. Values are mean ± standard error of the mean for n = 3 biological replicates. Values for the same specie in the same row with the same superscript letter (a to c) did not differ significantly (p > 0.05). Values for mucic acid concentration in the same column with the same superscript letter (A to C) did not differ significantly (p > 0.05)

The two marine *Trichoderma* transformants performed similarly (p > 0.05) under all conditions tested except with glycerol as co-substrate (Fig. 1; Table 1). The amount of mucic acid produced and of d-galacturonic acid utilized were dependent on the co-substrate (Fig. 1). Significantly more (p < 0.05) mucic acid (7.7 to 8.0 g L^−1^ mucic acid) was produced with lactose or d-xylose as co-substrate, rather than with d-glucose (6.6 g L^−1^) or glycerol (5.3 or 6.4 g L^−1^, Table 1). The marine *Trichoderma* transformants produced significantly more (p < 0.05) mucic acid during 6 days of incubation than *T. reesei* D-161646 in each condition tested (Table 1).

For the marine *Trichoderma* transformants T2 and T3, the calculated yield of mucic acid on d-galacturonic acid utilized (1.13 to 1.29 g g^−1^, Table 1) was above the theoretical yield of 1.08 g g^−1^, except for T3 with glycerol as co-substrate, indicating that the d-galacturonic acid concentrations at the end of the cultivations had been overestimated. When concentrations of d-galacturonic acid less than 2 g L^−1^ were assumed to be zero, the calculated yields were between 0.98 and 1.10 g g^−1^d-galacturonic acid utilized. The yield of mucic acid on d-galacturonic acid was independent of the co-substrate used.

Unlike the marine *Trichoderma* strains, *T. reesei* D-161646 produced significantly more (p < 0.05) mucic acid (5.4 ± 0.2 g L^−1^) with lactose as co-substrate than with d-xylose or glycerol and significantly less (p < 0.05) with d-glucose (2.8 ± 0.2 g L^−1^) than with other substrates (Table 1). The theoretical yield was only observed with d-xylose as co-substrate (Table 1).

The three *Coniochaeta* transformants each produced different amounts of mucic acid (p < 0.05), with strain C1 producing the most (3 to 5 g L^−1^) under all conditions, and C3 the least (1.1 g L^−1^; Table 1, Fig. 2). d-Galacturonate was generally utilized within less than 96 h (less than 72 h for the parental strain) and maximum mucic acid production was also observed at this time. Mucic acid concentrations remained constant (p > 0.05) after d-galacturonic acid had been utilized. To further assess whether or not *Conciochaeta* sp. MF729 or its transformants were able to use mucic acid as a substrate, they were also grown in the presence of added mucic acid with d-xylose or d-glucose as co-substrate, but no mucic acid was consumed [28]. Transformants C2 (2.4 g L^−1^) and C3 (1.1 g L^−1^) produced the same (p > 0.05) amount of mucic acid, regardless of the co-substrate, but C1 produced significantly less (p < 0.05) mucic acid with d-xylose as co- substrate (3.4 g L^−1^), compared to d-glucose (5.1 g L^−1^) or glycerol (4.5 g L^−1^).

Even though all d-galacturonic acid was utilized by the *Coniochaeta* strains, even transformant C1 produced less mucic acid than the *Trichoderma* transformants; significantly less (p < 0.05) in medium with glycerol or d-xylose as co-substrate, and slightly, but not significantly, less (5.1 ± 0.4 g L^−1^ compared to 6.5 g L^−1^, Table 1) with d-glucose as co-substrate. The yield of mucic acid on d-galacturonic acid was lower for the *Coniochaeta* transformants than for the *Trichoderma* transformants and always below the theoretical (Table 1). The highest yield observed with *Coniochaeta* transformants was 0.82 ± 0.05 g g^−1^ for C1 grown with d-glucose as co-substrate. Transformant C1 had higher (p < 0.05) yields than either C2 or C3.

The parental, non-modified LF328 and MF729 consumed d-galacturonic acid faster than the modified strains (Figs. 1 and 2).

### Production of mucic acid from poly-d-galacturonic acid in 24-well plates

The ability of transformants to produce mucic acid from poly-d-galacturonic acid was tested in 24-well plate cultivations with d-xylose or lactose as co-substrates for T2 and T3 and d-glucose as co-substrate for C1 and C2, based on the results with d-galacturonic acid as substrate [28]. *Coniochaeta* C3 was not included in these cultures since it was unable to produce more than 1.2 g L^−1^ mucic acid. The d-galacturonic acid content in poly-d-galacturonic acid media was measured after enzymatic hydrolysis in the absence of microorganisms, to determine the maximum d-galacturonic acid available in the experiment, and was observed to be 8.6 ± 0.1 g L^−1^ in the lactose medium, 9.1 ± 0.2 g L^−1^ in the d-xylose medium and 12.1 ± 1.1 g L^−1^ in the d-glucose medium. Because of the small culture volume in the 24-well plates the amount of unhydrolyzed polymer that remained as the cultivations progressed was not determined. The d-galacturonic acid present in the medium at the start of the cultivations defined the maximum possible mucic acid production.

**Fig. 3:**
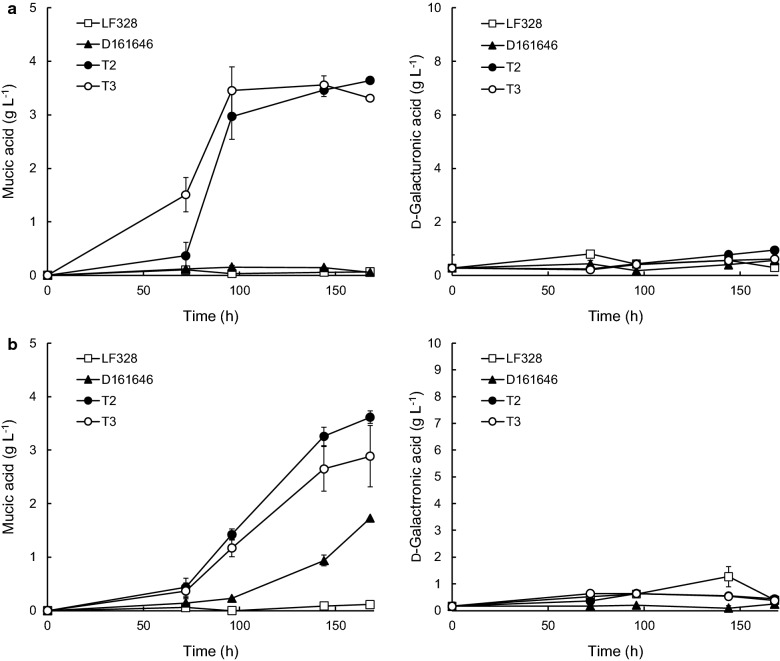
Mucic and residual d-galacturonic acid concentrations in 24-well plate *Trichoderma* cultures with poly-d-galacturonic acid. *Trichoderma* sp. LF328 and its transformants T2 and T3 were grown in 4 ml medium containing poly-d-galacturonic acid and lactose (**a**) or d-xylose (**b**) as co-substrate. *T. reesei Δgar1 udh* strain D-161646 was included as a positive control. Error bars represent ± standard error of the mean for n = 2 or 3 biological replicates

**Fig. 4:**
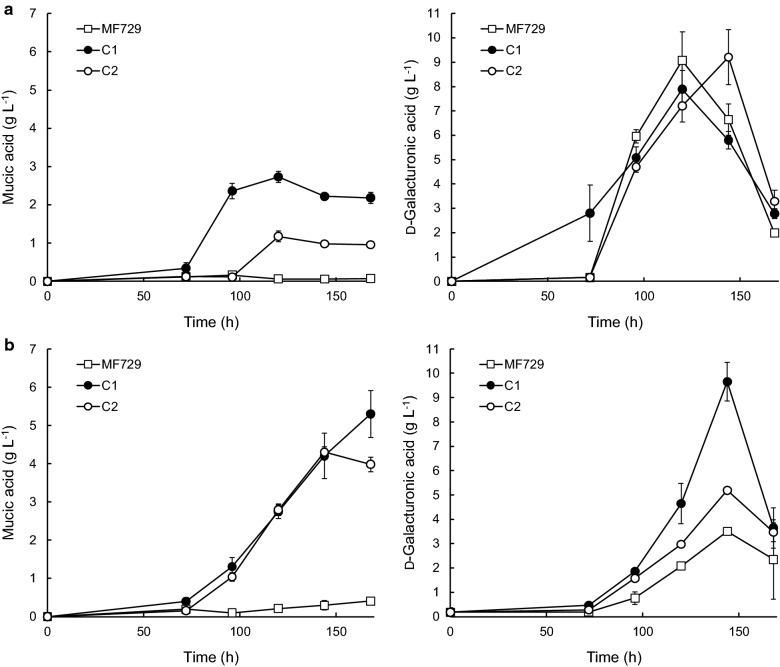
Mucic and residual d-galacturonic acid concentrations in 24-well plate *Coniochaeta* cultures with poly-d-galacturonic acid or pectin. *Coniochaeta* sp. MF729 and its transformants C1 and C2 were grown in 4 ml medium containing poly-d-galacturonic acid (**a**) or pectin (**b**) with d-glucose as co-substrate. Error bars represent ± standard error of the mean for n = 2 or 3

All transformants produced some mucic acid from poly-d-galacturonic acid during 7 day cultivations (Figs. 3 and 4; Tables 2 and 3). D-161646 produced mucic acid from poly-d-galacturonic acid with lactose as co-substrate, but not with d-xylose (Table 2). The marine *Trichoderma* transformants T2 and T3 produced similar (p > 0.05) amounts of mucic acid (2.9 to 3.6 g L^−1^, Table 2), and more (p < 0.05) than D-161646 (1.7 g L^−1^). Mucic acid production by T2 and T3 was the same (p > 0.05) with either lactose or d-xylose as co-substrate. There was no accumulation of d-galacturonic acid in the culture supernatant (Fig. 3) and it is unlikely that the poly-d-galacturonic acid was fully hydrolyzed. None-the-less, 34 to 42% of the d-galacturonic acid available in the poly-d-galacturonic acid was converted into mucic acid.

**Table 2:** Mucic acid production from poly-d-galacturonic acid by marine *Trichoderma* sp. LF328 transformants

Strain	T2	T3	D-161646
Lactose + poly-d-galacturonate			
Mucic acid (g L^−1^)	3.6 ± 0.1^a^	2.9 ± 0.6^a^	1.7 ± 0.0^b^
Conversion mucic acid/available d-galacturonic acid (g g^−1^)	0.42 ± 0.01	0.34 ± 0.07	0.20 ± 0.01
d-Xylose + poly-d-galacturonate			
Mucic acid (g L^−1^)	3.6^a^	3.6 ± 0.2^a^	0.1^b^
Conversion mucic acid/available d-galacturonic acid (g g^−1^)	0.40	0.39	0.01

*Trichoderma* sp. LF328 transformants were grown in 24-well plates using poly-d-galacturonic acid as substrate and either lactose or d-xylose as co-substrate. *T. reesei Δgar1 udh* strain D-161646 was included as a positive control. Values are mean ± standard error of the mean for n = 2 (D-161646) or 3 biological replicates. Values for mucic acid concentration in the same row with the same superscript letter (a or b) did not differ significantly (p > 0.05)

**Table 3:** Mucic acid production from poly-d-galacturonic acid or pectin by marine *Coniochaeta* sp. MF729 transformants

Strain	C1	C2
d-Glucose + pectin		
Mucic acid (g L^−1^)	5.3 ± 0.6^a^	4.2 ± 0.1^a^
Conversion mucic acid/available d-galacturonic acid (g g^−1^)	0.40	0.33
d-Glucose + poly-d-galacturonate		
Mucic acid (g L^−1^)	2.7 ± 0.2^a^	1.2 ± 0.1^b^
Conversion mucic acid/available d-galacturonic acid (g g^−1^)	0.23	0.10

*Coniochaeta* sp. MF729 transformants were grown in 24-well plates using poly-d-galacturonic acid or pectin as substrate with d-glucose as co-substrate. Values are mean ± standard error of the mean for n = 3 biological replicates. Values for mucic acid concentration in the same row with the same superscript letter (a or b) did not differ significantly (p > 0.05)

Both *Coniochaeta* C1 and C2 produced less (p < 0.05, student *t* test) mucic acid from poly-d-galacturonic acid than from d-galacturonic acid, with d-glucose as co-substrate. Substantial hydrolysis of the poly-d-galacturonic acid occurred, since d-galacturonic acid accumulated in the culture supernatant up until 120 or 144 h (Fig. 4). Mucic acid production was also maximal at 120 h, and was not associated with the subsequent disappearance of d-galacturonate from the supernatant. C1 converted 23% of the d-galacturonic acid available in the poly-d-galacturonic acid into mucic acid (Table 3). If one assumes that the poly-d-galacturonic acid was fully hydrolyzed at the time when residual d-galacturonic acid concentrations decreased in the culture supernatant, the approximate yield of mucic acid on utilized poly-d-galacturonic acid was 0.70 g g^−1^ at the time of maximal production for C1 and 0.50 g g^−1^ for C2.

### Production of mucic acid from pectin in 24-well plates

When *Trichoderma* LF328, T2, T3 or D-161646 were grown in 24-well plates with citrus pectin as substrate and either lactose or d-xylose as co-substrate, no mucic acid was produced, nor did d-galacturonic acid accumulate in the supernatant [28].

In contrast, *Coniochaeta* MF729, C1 and C2 hydrolyzed pectin to release d-galacturonic acid, which accumulated in the supernatant for up to 6 days, after which the amount decreased (Fig. 4) [28]. Both transformants C1 (5.3 ± 0.6 g L^−1^) and C2 (4.3 ± 0.1 g L^−1^) produced mucic acid from pectin (Fig. 4, Table 3).

### Production of mucic acid in pH-controlled bioreactor cultivations

The production of mucic acid by the new transformants was validated in bioreactor cultivations using conditions based on those described for *T. reesei* D161646 [20], but with a higher concentration of d-galacturonic acid in the feed for T2 since it utilized all d-galacturonic acid in the 24-well plates and D161646 did not. Pectin batch cultures were also assessed for *Conciochaeta* sp. MF729 C1, since it had produced equally well from D-galacturonic acid and pectin in the 24-well plate cultivations.

**Fig. 5:**
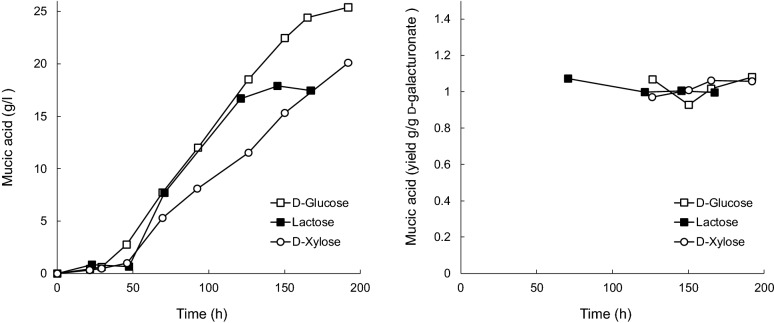
Mucic acid titer (left) and yield (right) in T2 fed-batch cultures. *Trichoderma* sp. LF328 T2 was grown at pH 4, 30 °C on d-galacturonic acid with d-glucose (22 g L^−1^), lactose (13 g L^−1^) or d-xylose (14 g L^−1^) as co-substrate. The feed contained 45 (lactose) or 67 (d-glucose and d-xylose) g L^−1^d-galacturonic acid. Values are adjusted for evaporation (~ 10%) which occurred during the fermentations

Trichoderma LF328 T2 produced up to 25 g L^−1^ mucic acid from d-galacturonic acid in fed-batch culture at pH 4 with d-glucose as co-substrate (Fig. 5) [28]. With d-xylose or lactose as co-substrates, 20 and 18 g L^−1^ mucic acid were produced, respectively (Fig. 5). The lactose culture however, had a lower concentration of d-galacturonate in the feed than the d-glucose and d-xylose cultures and there was no residual d-galacturonate in the culture supernatant at the end of the culture. The initial production rates on d-glucose (0.21 g L^−1^ h^−1^) and lactose (0.23 g L^−1^ h^−1^) were comparable. In contrast, the culture with d-xylose as co-substrate had residual d-galacturonate throughout the culture and the initial production rate was 0.17 g L^−1^ h^−1^. The yield of mucic acid on d-galacturonic acid was 1.02, 1.02 and 1.04 g [g d-galacturonic acid utilized]^−1^ with d-glucose, lactose and d-xylose as co-substrates, respectively (Fig. 5).

*Coniochaeta* MF729 C1 produced only 2 g L^−1^ mucic acid in fed-batch culture with d-glucose as co-substrate at pH 4 [28]. With pectin as substrate, 5 and 6 g L^−1^ mucic acid were produced at pH 5 and pH 4, respectively [28]. d-Galacturonic acid was not detected in the supernatant of pectin-fed cultures and remained less than 0.6 g L^−1^ in d-galacturonate fed cultures.

### Characterization of poly-d-galacturonase activity from *Trichoderma* LF328

**Fig. 6:**
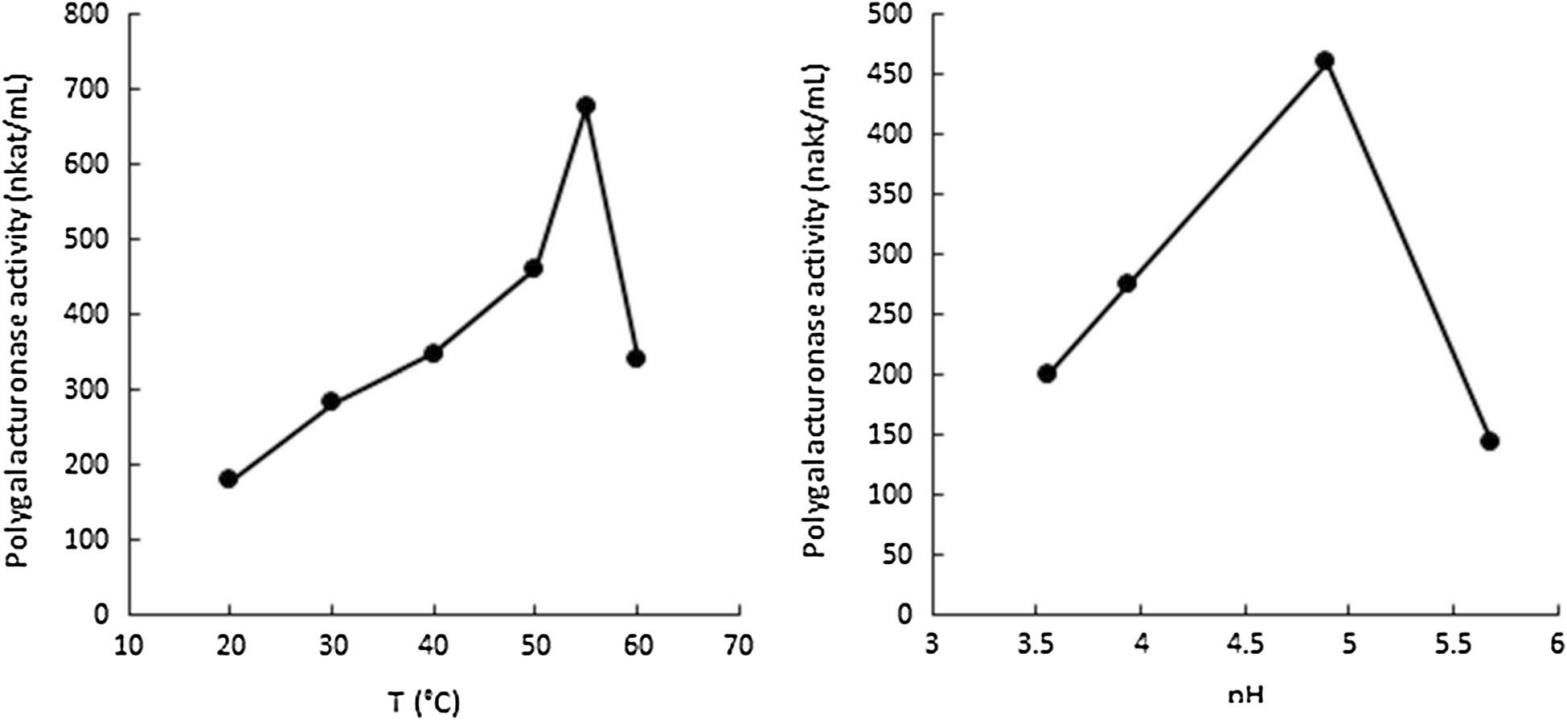
The effect of temperature and pH on the *Trichoderma* LF328 poly-d-galacturonase activity. Culture supernatant of *Trichoderma* LF328 was analyzed after 4 days cultivation on pectin at 28 °C, 200 rpm

LF328, pre-grown on d-xylose-containing medium, produced maximum poly-d-galacturonase activity after 4 to 7 days cultivation in flasks containing citrus pectin. The poly-d-galacturonase was most active at 55 °C and pH 4.9 (Fig. 6). Activity was lost within less than 15 min when incubated at 50 or 55 °C, but was stable for more than an hour when incubated at 40 °C.

No pectin lyase or pectate lyase activity was observed. No pectin lyase, pectate lyase or poly-d-galacturonase activity was measurable from MF729 cultures pre-grown on d-xylose and incubated with citrus pectin.

## Discussion

Gene amplification with degenerative primers revealed that *Trichoderma* sp. LF328 and *Coniochaeta* sp. MF729 possess a *gar2* gene but not *gar1*. In contrast, *T. reesei*, *A. niger* and *B. cinerea* each have both *gar1* and *gar2* genes [24, 25, 29]. In *T. reesei*, *gar1* encodes the functional d-galacturonate reductase [26] and *gar2* a putative reductase [24], the function of which is unknown. In *T. reesei*, deletion of *gar1* is sufficient to prevent growth on d-galacturonate, indicating that this is the only pathway in d-galacturonate catabolism [8]. In *A. niger* the main functional d-galacturonate reductase is encoded by *gaaA*, which is the orthologous gene for *gar2* of *T. reesei* [24]. Thus, different reductases are responsible in *T. reesei* and in *A. niger* for the reduction of d-galacturonic acid. In *B. cinerea*, both *gar1* and *gar2* encode functional reductases and are involved in the first step of the reductive pathway for d-galacturonic acid utilization and both genes must be deleted to inactivate the d-galacturonic acid reductive pathway [25]. Results of the present study showed that the functional d-galacturonate reductase differs in *Trichoderma* species, in *T. reesei* being encoded by *gar1* and in LF328 by *gar2*. In marine *Trichoderma* sp. LF328, disruption of *gar2* and expression of *udh* resulted in equimolar conversion of d-galacturonic acid to mucic acid, suggesting that *gar2* is the only functional d-galacturonic acid reductase in LF328: no d-galacturonic acid was diverted to the endogenous d-galacturonic acid pathway and all was thus available to the uronate dehydrogenase.

For *Coniochaeta* sp. MF729, disruption of *gar2* and expression of *udh* did not result in equimolar conversion of d-galacturonic acid to mucic acid (Tables 1 and 3), and d-galacturonic acid was sometimes consumed without production of mucic acid (Fig. 4). Another functional d-galacturonic acid reductase, not identified in the present study, may be present in MF729, explaining the low yield of mucic acid from d-galacturonic acid. Further study would be needed to understand the pathway in MF729. Elucidation of potential candidate genes for additional modifications could involve whole genome sequencing or an RNA expression analysis. Unlike *A. niger* [8, 9], MF729 did not metabolize mucic acid [28].

This study demonstrated that CRISPR-Cas9 and the universal SES-B were both functional in the two marine fungi engineered. Although short flanks were used in the homologous arms, correct integration was observed. Previously Liu et al. [30] observed that 0.2 kb flanks were sufficient to achieve homologous integration stimulated by CRISPR-Cas9, whereas Kuivanen et al. [31] did not obtain correct integrants when 0.1 kb flanks were used. The flanks used here were all greater than 0.1 kb, but in some cases less than 0.2 kb (i.e. 0.13 kb for one 5′ flank and 0.18 kb for one 3′ flank).

The marine *Trichoderma* transformants T2 and T3 produced the same (p > 0.05) amount of mucic acid, at similar yields in all conditions except production with glycerol as co-substrate, and can be considered homologous clones (i.e. the *udh* was correctly integrated in the genome, disrupting the *gar2*, with no other alterations to the genome). The relatively low titers for mucic acid production observed for D-161646 in the 24-well plates reported here, compared to those reported by [20], probably reflect the lower initial d-galacturonic acid concentration in the medium and the use of co-substrates other than lactose. Barth and Wiebe [22] reported that mucic acid production by D-161646 was higher with lactose than with d-glucose as co-substrate and production with d-xylose or glycerol had not previously been reported. D-161646 was used as a positive control for mucic acid production and the reason for the low amounts produced in these conditions was not explored further. The 24-well plates suggested that the marine *Trichoderma* transformants had the potential to produce more mucic acid, at equal or higher rates, than D-161646, as confirmed in bioreactor cultivation, in which 25 g L^−1^ mucic acid was produced (Fig. 5), compared to 21 g L^−1^ with D-161646 [20]. It should be noted that higher d-galacturonic acid concentrations were used in the feed with T2 than with D-161646, since the 24-well plates had indicated it could produce more mucic acid than D-161646, and that the feed concentration may have limited the maximum production by D-161646 in earlier studies.

The production of more mucic acid by marine *Trichoderma* transformants compared to D-161646 could result from the use of different promoters for the *udh* gene. In D-161646 the *udh* gene was under the control of a heterologous, constitutive promoter, *gpdA*, and in marine *Trichoderma* under the control of SES-B. The test conditions were developed for D-161646, but were expected to be suitable for the growth-associated SES-B promoter. Expression levels with SES promoters are comparable to, or stronger than, those of strong, endogenous promoters such as the transcription elongation factor (*TEF*), *gpdA* in *A. niger*, and *cbh1* in *T. reesei* [27]. The *cbh1* and *gpdA* promoters supported similar production levels of mucic acid in *T. reesei* [20], and SES-B was expected to support similar, rather than higher production levels. The high mucic acid titers of the marine *Trichoderma* transformants also reflect characteristics of the marine strain. Factors affecting its salt tolerance may confer tolerance to intracellular acid production, or it may have more efficient uptake of d-galacturonate than *T. reesei* D-161646, particularly at low substrate concentration.

Marine *Trichoderma* transformants produced mucic acid equally well with either lactose (7.7–8.0 g L^−1^) or d-xylose (7.8–7.9 g L^−1^) as co-substrates in small-scale batch cultures and with d-glucose and lactose in fed-batch cultures (Fig. 5), whereas *T. reesei* D-161646 preferred lactose (Table 1). Glucose appeared to repress mucic acid production in D-161646, whereas production by the marine transformants was similar with d-glucose or glycerol as co-substrate (Table 1). It has been shown that uptake of d-galacturonic acid can be repressed by the presence of other substrates [32, 33]. Co-substrates, d-xylose, glycerol, d-glucose or lactose, were generally consumed by *Trichoderma* strains before sampling started at 72 h, which should also have relieved initial repression effects.

The three *Coniochaeta* transformants each produced different (p < 0.05) amounts of mucic acid (Table 1, Fig. 2), suggesting that they differed in how the *udh* gene had been integrated into the genome. In the preliminary PCR screening of transformants, C2 had shown a different band pattern than C1 or C3, with the *gar2* locus possibly being rearranged, rather than deleted, supporting the hypothesis that different integration events had occurred in the three strains. In addition, although the *gar2* site was disrupted in C1 and C3, one or more additional copies of the expression cassette may have integrated elsewhere in the genome by nonhomologous end joining, which is known to be efficient in filamentous fungi. Integration of an extra copy at an alternative locus could explain why transformant C1 produced more mucic acid than either C2 or C3. However, since even C1 lost carbon from d-galacturonate to an alternative pathway, this was not investigated further.

*Coniochaeta* transformant C1 produced more mucic acid with d-glucose as co-substrate, than with either d-xylose or glycerol. This may suggest that with a preferred carbon source available, more d-galacturonic acid was channeled to the *udh* pathway, or that d-glucose repressed the putative alternative pathway. Transformants C2 and C3 were not affected by the type of co-substrate, producing the same amount in all conditions (Table 1).

Both *Trichoderma* LF328 (0.05 ± 0.001 h^−1^) and *Coniochaeta* MF729 (0.06 ± 0.003 h^−1^) were selected for expression of *udh* because they grew on d-galacturonic acid and pectin in earlier MTP screening [7]. When provided with citrus pectin, however, it was clear that LF328 was unable to hydrolyze it, whereas it could hydrolyze poly-d-galacturonate and produced mucic acid from it (Fig. 4). *T. reesei* is known to have genes from the GH28 family (including poly-d-galacturonases) of hydrolytic enzymes, but not other types of pectinase [29], and D-161646 was not expected to hydrolyze pectin. Marine *Trichoderma* LF328 similarly appears to produce poly-d-galacturonases only. T2 and T3 produced 3.6 g L^−1^ mucic acid from poly-d-galacturonic acid, with essentially no accumulation of d-galacturonic acid in the supernatant, indicating that the uronate dehydrogenase was able to catalyze the conversion at a rate equivalent to the hydrolysis of the polymer. That less than half the d-galacturonic acid available in the polymer was converted into mucic acid suggests that the hydrolysis was not complete. The poly-d-galacturonase(s) activity was probably low and potentially inefficient (temperature optimum 55 °C, pH optimum 4.9) in the conditions used for growth (28 °C and initial pH 5.5). Addition of commercial pectinase would be needed to obtain good mucic acid production from pectin or poly-d-galacturonic acid with the marine *Trichoderma* transformants, as with D-161646 [20].

*Coniochaeta* strains degraded both poly-d-galacturonate and pectin, and d-galacturonic acid accumulated in the culture supernatant of both parent and transformants (Fig. 4). Thus *Coniochaeta*’s pectinases were hydrolyzing the polymers at higher rates than the cells could take up the d-galacturonic acid or convert it to mucic acid. Concentrations of 8–9 g L^−1^d-galacturonic acid were observed in the supernatant of poly-d-galacturonic acid cultures before concentrations started to decrease, suggesting that most if not all of the poly-d-galacturonic acid had been degraded. Pectin appeared to be similarly well degraded, although the maximum d-galacturonic acid in the supernatant of MF729 and C2 cultures was less than from poly-d-galacturonic acid. Neither poly-d-galacturonase nor pectin/pectate lyase activity was detectable in flask cultures of MF729, indicating that pectinases were not efficiently produced in defined medium or that the assay conditions required optimizing. Conditions were not explored further since the production of mucic acid was low.

Although similar amounts of d-galacturonic acid were provided from poly-d-galacturonic acid (12 ± 1 g L^−1^) and pectin (13 ± 1 g L^−1^), *Coniochaeta* C1 and C2 produced significantly (p < 0.05) less mucic acid from poly-d-galacturonic acid than from pectin (Table 3 or Fig. 4). Surprisingly, C2 produced a similar (p > 0.05) amount of mucic acid (4.3 ± 0.1 g L^−1^) as C1 (5.3 ± 0.6 g L^−1^) with pectin as the substrate, whereas in all other media it produced significantly less (p < 0.05). These results suggest that the additional carbon sources provided from pectin (e.g. d-xylose, l-rhamnose) enabled more carbon from d-galacturonic acid to be channeled to mucic acid, rather than the alternative pathway. Pectin was thus a good substrate for mucic acid production with *Coniochaeta*, and could be used without addition of commercial enzymes. None-the-less, it would be necessary to completely disrupt other routes of d-galacturonic acid utilization before *Coniochaeta* MF729 could be used for mucic acid production. Production from pectin remained low (6 g L^−1^) in pH-controlled bioreactor culture, even though more pectin was added than in the 24-well plates A consolidated process for conversion of pectin to mucic acid was developed with *A. niger*, another fungus that naturally degrades pectin, and resulted in conversion of about 30% of available d-galacturonic acid to mucic acid [9].

## Conclusions

The results presented here demonstrate the usefulness of the SES-B system to engineer two novel marine fungi for production of mucic acid. Both marine species were successfully engineered using CRISPR-Cas9 and the synthetic expression system was functional in both species. Although the *Coniochaeta* sp. transformants produced mucic acid directly from pectin, the metabolism of d-galacturonic acid was not completely disrupted and mucic acid amounts were low. However, the pathway was completely disrupted in transformants of the marine *Trichoderma* sp. and these produced 16% more mucic acid (25 g L^−1^) than a previously constructed *T. reesei* mucic acid producing strain (21 g L^−1^, [20]), when grown under similar conditions. This demonstrates that marine fungi may be useful as production organisms, not only for native enzymes or bioactive compounds, but also for other compounds.

## Materials and methods

### Strains

The marine fungi *Coniochaeta* sp. MF729 and *Trichoderma* sp. LF328 were obtained from the GEOMAR Helmholtz Centre for Ocean Research Kiel, Germany. *Trichoderma reesei* QM6a ∆*gar*1 *udh* (VTT D-161646, VTT Culture Collection) was used as a positive control in cultivations. *T. reesei* QM6a (VTT D-071262T, ATCC13631, VTT Culture Collection) was used as a control in PCR. *Escherichia coli* TOP10 (Invitrogen) was used to propagate plasmids. *Saccharomyces cerevisiae* FY834 [34] was used for homologous recombination in the construction of plasmids containing expression cassettes.

### Amplification of d-galacturonate reductase genes *gar1* and *gar2* from LF328 and MF729

Conserved regions were observed in *gar1* sequences when those from available fungal species were aligned: the highly conserved regions in the 5´ and the 3′ ends of the gene were selected for the *gar1* forward and reverse primer sites, respectively. Similarly, alignment of *gar2* sequences showed the presence of conserved regions and degenerative primers were designed to the 5′ and 3′ prime end conserved sequences of *gar2* genes. The following sequences were used for design of primers. For *gar1*, *Trichoderma reesei gar1* (accession number AY862503, GenBank), *Aspergillus niger* NADPH-dependent reductase gene, putative *gar1* (accession number AM270369, An16g04770, GenBank) and *Botrytis cinerea* Bc*gar1* (accession number XM_001559172, GenBank). These *gar1* sequences were aligned using the EMBL-EBI Multiple Sequence Alignment alignment tool [35]. Primers were designed to homologous regions of the aligned sequences. The *gar1* degenerative primer mix contained one-third of each primer designed, based on the *T. reesei*, *A. niger* or *B. cinerea* sequence (see Additional file 1: Table S1). For g*ar2*, four different sequences were selected: *T. reesei gar2* (accession number EF563987, GenBank), *A. niger gaaA* (accession number EF563988, GenBank), *B. cinerea* Bc*gar2* (XM_024691756, GenBank) and yeast, *Naganishia diffluens*d-galacturonic acid reductase gene (accession number HV538330, GenBank). The *gar2* degenerative primer mix contained one-fourth of each primer, based on *T. reesei*, *A. niger*, *B. cinerea* and *N. diffluens* sequence. PCRs were performed using the Phire Plant Direct PCR Kit (Thermo Scientific) according to the manufacturer’s instructions. The PCR reaction was performed as gradient-PCR to enable binding of the primers even if there were some mismatches to the target sequence. *T. reesei* QM6a genomic DNA was used as a control in PCR reactions as it is known to contain both *gar1* and *gar2* genes.

### Construction of the *udh* expression cassette

A plasmid containing the *Agrobacterium tumefaciens udh* gene, codon optimized for *Aspergillus niger* [8], was used as template to amplify the *udh* ORF by PCR. The B8121 plasmid obtained from A. Rantasalo contained the whole SES-B universal system together with a NAT cassette for Nourseothricin (NAT) selection [27]. To construct the expression cassette for the present work, two fragments were digested from the B8121 plasmid: a fragment containing the synthetic SES-B promoter, digested with *Sgs*I and *Pac*I, and a fragment containing the rest of the SES-B together with the NAT selection cassette, digested with *Bam*HI and *Pae*I. The 5′ and 3′ flanks for targeting the cassette to the *gar2* locus were amplified by PCR using LF328 or MF729 chromosomal DNA as templates. The Phire Plant Direct PCR Kit (Thermo Scientific) or DreamTaq Polymerase (Thermo Scientific) were used for amplification of 5′ and 3′ flanks and the KAPA HiFi HotStart Kit (KAPA Biosystems) for amplification of the *udh* ORF. Each primer contained homologous overlapping ends (see Additional file 1: Table S1) to combine fragments prepared by PCR and those obtained with restriction enzyme digestions. Construction of the expression cassette was performed in a YEplac195 shuttle plasmid possessing genes for ampicillin and uracil selection [36]. The YEplac195 plasmid was digested with *Hind*III enzyme and the YEplac195 backbone was introduced into FY834 together with the fragments with overlapping ends, based on the protocol of Gietz and Woods [37]. *S*. *cerevisiae* was used to obtain plasmids obtained through homologous recombination. After amplification in *E. coli*, plasmids were checked by restriction enzyme digestion and sequencing.

### Strain generation

The expression plasmids were digested with *Bgl*II to release a linearized cassette. The marine *Trichoderma* sp. LF328 and *Coniochaeta* sp. MF729 were transformed with a cassette containing either LF328 or MF729 specific *gar2* flanks, together with ribonucleoprotein (RNP) complex. The three parts of the RNP complex (*Streptococcus pyogenes* Cas9 nuclease, tracrRNA and crRNA) were purchased from Integrated DNA Technologies (IDT, Iowa, USA). RNP assembly was carried out following the manufacturer’s instructions. In assembling the RNP complex, a chemically synthesized crRNA containing a 20 bp protospacer sequence for *gar2* was first hybridized with tracrRNA and then assembled with Cas9 protein in vitro. All the protospacer sequences (see Additional file 1: Table S1) in crRNAs were designed with IDT software [38]. RNP complexes were formed and used in the transformation reactions simultaneously. The break in *gar2* was repaired by the homology-directed repair (HDR) mechanism using the donor DNA.

All transformations were carried out using the protoplast transformation method of [39]. In the transformations, 50 µL protoplasts (1–2 × 10^8^ protoplast mL^−1^) were incubated with 4 µg cassette DNA and 15–30 nM RNP complex. Transformants were selected on minimal medium containing NAT. A relatively high concentration of NAT was needed to avoid background: for *Trichoderma* sp. 1000 µg mL^−1^ and for *Coniochaeta* sp. 500 µg mL^−1^. The isolates were screened by PCR with primers listed in Additional file 1 (see Additional file 1: Table S1) to verify the presence of the expression cassette and disruption of the *gar2* gene. Single spore isolates were obtained for two *Trichoderma* sp. and three *Coniochaeta* sp. transformants, which were tested further in 4 mL cultivations.

### Media

Low phosphate medium [22] was used for 24-well plate and bioreactor cultivations, with variations in the concentration and source of d-galacturonate and the co-substrates. Cultivations were provided 7.1 to 7.9 g L^−1^d-galacturonate (monohydrate, Sigma), 12 g L^−1^ poly-d-galacturonate (Fluka, providing 8.8 ± 0.1 g L^−1^d-galacturonate) or 20 g/l citrus peel pectin (poly-d-galacturonic acid methyl ester, Sigma, providing 13.3 ± 0.4 g L^−1^ poly-d-galacturonate) for mucic acid production. d-Glucose (11.0 ± 0.8 g L^−1^), lactose (9.5 ± 0.3 g L^−1^), d-xylose (10.4 ± 0.6 g L^−1^) or glycerol (11.4 ± 0.1 g L^−1^) were added as co-substrate. All 24-well plate media contained 100 mM 1,4-piperazinedipropanesulfonic acid (PIPPS) and the pH was adjusted to 5.5 with NaOH. To assess the ability of *Coniochaeta* strains to consume mucic acid, poly-d-galacturonate was replaced with 10 g L^−1^ mucic acid in medium containing 5 g L^−1^d-glucose or d-xylose as co-substrate [9].

The same medium, lacking PIPPS, was used for fed-batch bioreactor cultivation, with 4 to 5 g L^−1^d-galacturonate and 21 g L^−1^ lactose, 21 g L^−1^d-xylose or 23 g L^−1^d-glucose in the batch phase [22] and 45 (lactose as co-substrate) or 67 (d-xylose and d-glucose as co-substrates) g L^−1^d-galacturonate in the feed, with 13 g L^−1^ lactose, 14 g L^−1^d-xylose or 23 g L^−1^d-glucose. For *Coniochaeta* MF729 C1, the feed contained 23 g L^−1^d-galacturonic acid with 12 g L^−1^d-glucose, or d-galacturonic acid was replaced with pectin (45 g L^−1^, providing ~ 25 g L^−1^d-galacturonic acid). Both the batch medium and the feed also contained 2 g L^−1^ yeast extract. NaOH (2 M) was used to maintain constant pH at 4 or 5.

Medium for enzyme producing flask cultures was prepared using Yeast Nitrogen Base without amino acids (Difco™), with carbon provided as 10 g L^−1^d-xylose, 10 g L^−1^ pectin from citrus (Fluka Biochemika) and 1 g L^−1^ yeast extract in pre-cultures and 15 g L^−1^ citrus pectin without d-xylose or yeast extract for production of hydrolytic enzymes. The pH of the medium was adjusted to 5 for pre-cultures and to 4.2 for enzyme production.

*E. coli* was grown in Luria Broth culture medium supplemented with 100 µg ml^−1^ ampicillin. The yeast FY834 was grown in YP-medium (10 g yeast extract L^−1^ and 20 g peptone L^−1^) supplemented with 20 g d-glucose L^−1^ or in uracil deficient synthetic complete medium [40] supplemented with 20 g d-glucose L^−1^ (SCD-URA) for uracil auxotrophic selection after transformation.

### Culture conditions

Twenty-four-well plates (Whatman 10 mL round bottom Uniplate) contained 4 mL medium per well. Wells were inoculated with approximately 10^6^ spores (2.5 × 10^5^ spores mL^−1^ final concentration). For each medium, three replicate wells (biological replicates) were inoculated with each transformant (expressing *udh* under the control of SES), three wells with non-modified original strains (negative control), and three wells with D-161646 (positive control). Two or three wells for each medium were left without inoculum (technical replicates) and were used as a control to assess evaporation from the wells. The plates were covered with adhesive, breathable rayon fiber film for culture plates (VWR) and incubated at 28 °C, 800 rpm (Infors HT Microtron), for up to 7 days. Samples (100–200 µL) were taken daily after 72 h using wide-mouth 1000 µl pipette tips, and stored at − 20 °C. The maximum d-galacturonic acid concentration that could be digested from poly-d-galacturonic acid and pectin containing media was evaluated by enzymatic treatment using Pectinex Ultra SP-L (1 ml L^−1^, Novozymes) and Pectinex Smash (0.1 ml L^−1^, Novozymes) at +43˚C for 20 h.

Flasks for enzyme production were incubated at 28 °C, 200 rpm. Pre-culture flasks (100 mL containing 20 mL medium) were incubated for 3 days, then biomass from 12 mL culture was collected by centrifugation to remove excess d-xylose and re-suspended in 40 mL enzyme production medium in 250 mL flasks. Enzyme production flasks were incubated for up to 160 h, with maximal activity observed between 96 and 160 h. Culture supernatant was collected by centrifugation and stored at − 20 °C for analysis.

Braun Biostat B 2 L and Biostat Q 1 L reactors (Sartorius AG) were used for large-scale cultivations, with 1.5 and 0.5 L medium, respectively, in the batch phase. Cultures were agitated at 500 rpm (1 L) or 300–700 rpm (2 L) with approximately 0.6 volume gas (volume culture)^−1^ min^−1^. NaOH (2 M) was used to maintain constant culture pH (pH 4.0). *Trichoderma* LF328 T2 cultures were maintained at 35 °C, but *Coniochaeta* MF729 C1 cultures at 28 °C. Pre-cultures for bioreactor cultivations were prepared as described by [20].

*E. coli* was grown at 37˚C and 250 rpm. FY834 was cultivated at 30˚C with 250 rpm.

### Chemical analyses

The concentrations of d-glucose, lactose, d-galactose, d-galacturonic acid and mucic acid were determined by HPLC using a Fast Acid Analysis Column (100 mm × 7.8 mm, BioRad Laboratories) linked to an Aminex HPX-87H organic acid analysis column (300 mm x 7.8 mm, BioRad Laboratories) with 5 mM H_2_S0_4_ as eluent at a flow rate of 0.3 mL min^−1^. The column was kept at 55 °C. The detectors were Waters 410 Differential Refractometer and a Waters 2487 Dual Wavelength UV (210 nm) detector. Data acquisition was done using Waters Empower 3 software. Samples were diluted with eluent to give concentrations of mucic acid between 1 and 3 g L^−1^, then heated to 100 °C for 1 h to solubilize crystals of mucic acid and centrifuged at room temperature to remove biomass, prior to HPLC analysis, as described by [20]. Heating the samples in dilute acid results in conversion of a proportion of linear mucic acid to the lactone form, the retention time of which is close to that of d-galacturonic acid and can result in overlapping peaks when the lactone concentration is high. HPLC chromatograms were processed conservatively for d-galacturonic acid, assuming the tail of the galactarolactone peak would contain d-galacturonic acid. This method may over-estimate the presence of d-galacturonic at the end of some cultures. The retention time for PIPPS in this system resulted in peaks which overlap that of glycerol. Glycerol concentrations were estimated from the shoulder of the PIPPS peak using both area and height. Measurements of substrates in un-inoculated wells were used to adjust concentrations in inoculated wells of the same medium for evaporation [20]. Yields of product on substrate were calculated as the grams of mucic acid produced per gram of D-galacturonic acid utilized, based on the evaporation adjusted values.

### Enzyme analyses

Measurement of poly-d-galacturonase activity was based on the method of [41], with temperature and pH adjusted as indicated in the results. Thermostability was assessed by pre-incubating the samples in sodium citrate buffer for various intervals before assaying the remaining activity.

For pectin lyase activity, 5 g L^−1^ citrus pectin was dissolved in 25 mM sodium citrate buffer pH 5.5 and the solution filtered through Whatman^®^ No. 3 filter paper. Substrate (250 µL) was mixed with sample (125 µL) and incubated 10 min at 40 °C, after which absorbance was measured at 235 nm. Pectin lyase was assayed with and without the addition of 1 mM CaCl_2_. Pectate lyase was assayed using 5 g L^−1^ poly-d-galacturonic acid dissolved in Milli-Q^®^ water with the pH adjusted to 7.2 using NaOH. Substrate (500 µL) was mixed with 450 µL ammonium buffer (0.2 M, pH 9.5, containing 1 mM CaCl_2_) at 30 °C. Sample (50 µL) was added and incubated for 10 min, after which the reaction was terminated by addition of 1 mL sodium acetate buffer (0.2 M, pH 3.8). Absorbance was measured at 235 nm.

### Statistical analyses

Data are presented as mean ± standard error of the mean. Analysis of Variance was used for comparisons of three or more strains and to compare production in three or more media for one strain, with significant differences identified using Fisher’s multiple range test. The student-t test was used for comparisons of two strains or media.

## Supplementary information

**Additional file 1: Table S1.** List of primers used. **Figure S1.** The fungal pathway for d-galacturonic acid metabolism (reactions 1–4) and the reactions (5–6) necessary to produce mucic acid from d-galacturonic acid. The enzyme are: (1) d-galacturonate reductase EC 1.1.1.365, (2) l-galactonate dehydratase EC 4.2.1.146, (3) 2-keto-3-deoxy-galactonate aldolase EC 4.1.2.54 and (4) l-glyceraldehyde reductase EC 1.1.1.372, (5) d-galacturonate (uronate) dehydrogenase EC 1.1.1.203, (6) lactonase or spontaneous opening. (See Richard P, Hilditch S. d-Galacturonic acid catabolism in microorganisms and its biotechnological relevance. Appl Microbiol Biotechnol. 2009; 82:597–604 and Kuivanen J, Biz A, Richard P. Microbial hexuronate catabolism in biotechnology. AMB Expr 201; 9:16 for references and review of other pathways of d-galacturonic acid metabolism, including details on the enzymatic opening of the galactarolactone to the linear mucic acid, reaction 6.) **Figure S2.** The *gar2* sequences obtained for Trichoderma sp. LF328 and Coniochaeta sp. MF729 *gar2* gene.

## Data Availability

The datasets supporting the conclusions of this article are included within the article and/or are available in the Zenodo repository (https://zenodo.org/), 10.5281/zenodo.3696802 [28].
