# Supplementary material for: Engineering marine fungi for conversion of d-galacturonic acid to mucic acid
Source: Microb Cell Fact. 2020 Jul 31;19:156. doi: 10.1186/s12934-020-01411-3 (PMC7393721; doi:10.1186/s12934-020-01411-3)
Supplement: Supplementary file 1 — Additional file 1: Table S1. List of primers used. Figure S1. The fungal pathway for d-galacturonic acid metabolism (reactions 1–4) and the reactions (5–6) necessary to produce mucic acid from d-galacturonic acid. The enzyme are: (1) d-galacturonate reductase EC 1.1.1.365, (2) l-galactonate dehydratase EC 4.2.1.146, (3) 2-keto-3-deoxy-galactonate aldolase EC 4.1.2.54 and (4) l-glyceraldehyde reductase EC 1.1.1.372, (5) d-galacturonate (uronate) dehydrogenase EC 1.1.1.203, (6) lactonase or spontaneous opening. (See Richard P, Hilditch S. d-Galacturonic acid catabolism in microorganisms and its biotechnological relevance. Appl Microbiol Biotechnol. 2009; 82:597–604 and Kuivanen J, Biz A, Richard P. Microbial hexuronate catabolism in biotechnology. AMB Expr 201; 9:16 for references and review of other pathways of d-galacturonic acid metabolism, including details on the enzymatic opening of the galactarolactone to the linear mucic acid, reaction 6.) Figure S2. The gar2 sequences obtained for Trichoderma sp. LF328 and Coniochaeta sp. MF729 gar2 gene. [file 12934_2020_1411_MOESM1_ESM.docx]

Additional file 1

**Engineering marine fungi for conversion of d-galacturonic acid to galactaric acid**

Virve Vidgren*, Satu Halinen, Anu Tamminen, Susanna Olenius, Marilyn G. Wiebe

VTT Technical Research Centre of Finland Ltd, Tietotie 2, P.O. Box 1000, FI-02044 VTT, Finland

***Corresponding author**

VTT Technical Research Centre of Finland Ltd, Tietotie 2, P.O. Box 1000, FI-02044 VTT, Espoo, Finland

Tel: +358 50 531 23 14

Email: virve.vidgren@vtt.fi

**Table S1.** List of primers used.

**Figure S2.** The *gar2* sequences obtained for Trichoderma sp. LF328 and Coniochaeta sp. MF729 *gar2* gene

**Table S1.** List of primers used.

| **Description** | **Name** | **Sequence** | **Target gene** |
| --- | --- | --- | --- |
| Primers for cassette construction | udh_FRW | ACCTCTACAACTCCAATACACTTAATTAAAATGGCCATGAAGCGCCTG | *udh* in GUDH_A-tum plasmid |
|  | udh_REV | ATAATAAAAATCATAAATCATAAGAAATTCG GATC TCAGGACTGCTTGAAGATGG | *udh* in GUDH_A-tum plasmid |
|  | 5’_flank_FRW_328 | CAGGAAACAGCTATGACCATGATTACGCCAAGCTAGATCTGCCTCACCCTCTTCGACCTG | LF328 *gar2* |
|  | 5’_flank_REV_328 | TTGTTCCCTTTAGTGAGGGTTAATTCGGCGCGGGCGTCGGCATCGACCTT | LF328 *gar2* |
|  | 5’_flank_FRW_729 | CAGGAAACAGCTATGACCATGATTACGCCAAGCTAGATCTGGCGAGGCAAGGTCGGGC | MF729 *gar2* |
|  | 5’_flank_REV_729 | TTGTTCCCTTTAGTGAGGGTTAATTCGGCGCGGGGAACGAGTCGAACGAGGT | MF729 *gar2* |
|  | 3’_flank_FRW | TGTCTATGCCCTGCCCCTAATAGATGCATGGCCATCACCATCTTCACCCC | LF328 and MF729 *gar2* |
|  | 3’_flank_REV_328 | GATCCTCTAGAGTCGACCTGCAGGCATGCAAGATCTGCTTGTGGTGCTCGATGAAGA | LF328 *gar2* |
|  | 3’_flank_REV_729 | GATCCTCTAGAGTCGACCTGCAGGCATGCAAGATCTGCTTGTGGTGCTCGATGTAGA | MF729 *gar2* |
|  | SES_prom_REV | TTTAATTAAG TGTATTGGAGTTGTAGAGG | SES promoter |
|  | udh_REV | ATAATAAAAATCATAAATCATAAGAAATTCG GATC TCAGGACTGCTTGAAGATGG | *udh* |
| Primers for *gar1* and *gar2* amplification | gar1_An_FRW | GAGATCCCTGCTCTCGGACTCG | *A. niger putative gar1* |
|  | gar1_An_REV | GGGTGGTTCTCAATCTGGTTG | *A. niger putative gar1* |
|  | gar1_Bc_FRW | GAGATTCCGGCATTAGGACTCG | *B. cinerea gar1* |
|  | gar1_Bc_REV | GGGTGATTTTCAATTTGGTTGACG | *B. cinerea gar1* |
|  | gar1_Tr_FRW | GAGATTCCAGCTGTTGGTCTCGG | *T. reesei gar1* |
|  | gar1_Tr_REV | GGGTGGTTCTCAATCTGGTTGA | *T. reesei gar1* |
|  | gaaA_An_FRW | GGAACAGGCGAGTACACGACC | *A. niger gaaA* |
|  | gaaA_An_REV | GTAGGCCGGGTCGAAGCG | *A. niger gaaA* |
|  | gar2_Bc_FRW | GGAACCGGTGAATATACCACCG | *B. cinerea gar2* |
|  | gar2_Bc_REV | GTACGCGGGATCGAATCGTTT | *B. cinerea gar2* |
|  | gar2_Nd_FRW | GGTACCGGCGAGTACACCACC | *N. diffluens GAR2* |
|  | gar2_Nd_REV | GTAGGCCGGGTCGTACCG | *N. diffluens GAR2* |
|  | gar2_Hj_FRW | GGCACCGGCGAGTACACG | *T. reesei gar2* |
|  | gar2_Hj_REV | GTAGGCCGGGTCGAAGCG | *T. reesei gar2* |
| Protospacer sequences | crRNA LF328 | CTTCAATGAGCTTGTACGTACGG | LF328 *gar2* |
|  | crRNA MF729 | TCCCTGCTGACGTCGTTGGGCGG | MF729 *gar2* |
| Primers for transformant verification | SES_FRW | CGCGCCGAATTAACCCTCAC | SES promoter |
|  | udh_screen_FRW | ACCCCATCTTCAAGCAGTCCTG | udh |
|  | TEF_REV | GAACATCAGGTGTTGATGATGGGT | TEF1t |
|  | 328_screen_FRW | GACAAAAAGGTGGGCGTTGT | LF328 gar2 |
|  | 328_screen_REV | CTTGTAGGCCGGGTCGAA | LF328 gar2 |
|  | 729_screen_FRW | ACACGACCGGCTTCGTCGG | MF729 gar2 |
|  | 729_screen_REV | GTAGGCCGGGTCGAAGC | MF729 gar2 |

**Figure S2**. The fungal pathway for d-galacturonic acid metabolism (reactions 1-4) and the reactions (5-6) necessary to produce mucic acid from d-galacturonic acid. The enzyme are: (1) d-galacturonate reductase EC 1.1.1.365, (2) l-galactonate dehydratase EC 4.2.1.146, (3) 2-keto-3-deoxy-galactonate aldolase EC 4.1.2.54 and (4) l-glyceraldehyde reductase EC 1.1.1.372, (5) d-galacturonate (uronate) dehydrogenase EC 1.1.1.203, (6) lactonase or spontaneous opening. (See Richard P, Hilditch S. d-Galacturonic acid catabolism in microorganisms and its biotechnological relevance. Appl Microbiol Biotechnol. 2009; 82:597-604 and Kuivanen J, Biz A, Richard P. Microbial hexuronate catabolism in biotechnology. AMB Expr 201; 9:16 for references and review of other pathways of d-galacturonic acid metabolism, including details on the enzymatic opening of the galactarolactone to the linear mucic acid, reaction 6.)

**Figure S2**. The *gar2* sequences obtained for *Trichoderma* sp. LF328 and *Coniochaeta* sp. MF729 *gar2* gene

> *Trichoderma* sp. LF328 *gar2*

TGGGCGTTGTCGGCCTCACCCTCTTCGACCTGCGACGACGAGGCAAAGTCGGCCAGCTGGGCATGGTCGGCGTCAATGGCACCAAGTTTCCCGCAATCCGTACGTACAAGCTCATTGAAGATGCTCCCGGGCCAAAGTTGAGACTGACTGTGAACTCGCCGCATTGTAGGAGAGCACCTCAACAAGAACATCACCCAAGTCTACAACAACCTGGACACCTCGTTCGACTCATTCCCAGCCAACGACAAGGTCGATGCCGACGCCTACAAGGCCGCCATTGACCAGCTCAAGCCCGGCGATGCCATCACCATCTTCACCCCCGATCCTACCCACTTCCCCATTGCGCTGTACGCCATCGAGCGCGGCATCCACGTCCTCATCACTAAGCCCGCCGTGAAGCTGCTGGAGCACCACCTGGAGCTGGCCCAAAAGGCCGCCGAAAAGGGCGTCTACGTCTTCATCGAGCACCACAAGCAAATTCGACCCGGCCTACAAGCG

> *Coniochaeta* sp. MF729 *gar2*

AGTCGGCGTCGTCGGCCTGTCCATGTTCGACCTGCGGCGGCGAGGCAAGGTCGGCAAGCTGGGCATGGTCGGCACAAACGGCACAAAGTTCCCGGCCATCCGCGAGCACCTGCGCAAGAACATCCAGCAGGTCTACAACAACCTCGACACCTCGTTCGACTCGTTCCCGCCCAACGACGTCAGCAGGGACCCGGACTCGTACAAGGCCGCCATCGACAGCCTCGGCAAGGGCGACGCCATCACCATCTTCACCCCGGACACCACCCACTTCCCCATCGCCCTCTACGCCATCGAGCGCGGCGTCCACGTCATGATCACCAAGCCCGCCGTCAAGCTGCTCGAGCACCACCAGGCCCTCATCGACGCCGCCCGCACCCACGGCGTCTACGTCTACATCGAGCACCACAAGCGCTTCG
